# Zebrafish: A complete animal model to enumerate the nanoparticle toxicity

**DOI:** 10.1186/s12951-016-0217-6

**Published:** 2016-08-20

**Authors:** Chiranjib Chakraborty, Ashish Ranjan Sharma, Garima Sharma, Sang-Soo Lee

**Affiliations:** 1 Department of Bioinformatics, School of Computer and Information Sciences, Galgotias University, Greater Noida, Uttar Pradesh India; 2 Institute of Skeletal Aging and Orthopedic Surgery, Hallym University-Chuncheon Sacred Heart Hospital, Hallym University, Chuncheon, Gangwon-do 24252 Republic of Korea

**Keywords:** Zebrafish, Nanoparticle, Toxicity, Animal model

## Abstract

Presently, nanotechnology is a multi-trillion dollar business sector that covers a wide range of industries, such as medicine, electronics and chemistry. In the current era, the commercial transition of nanotechnology from research level to industrial level is stimulating the world’s total economic growth. However, commercialization of nanoparticles might offer possible risks once they are liberated in the environment. In recent years, the use of zebrafish (*Danio rerio*) as an established animal model system for nanoparticle toxicity assay is growing exponentially. In the current in-depth review, we discuss the recent research approaches employing adult zebrafish and their embryos for nanoparticle toxicity assessment. Different types of parameters are being discussed here which are used to evaluate nanoparticle toxicity such as hatching achievement rate, developmental malformation of organs, damage in gill and skin, abnormal behavior (movement impairment), immunotoxicity, genotoxicity or gene expression, neurotoxicity, endocrine system disruption, reproduction toxicity and finally mortality. Furthermore, we have also highlighted the toxic effect of different nanoparticles such as silver nanoparticle, gold nanoparticle, and metal oxide nanoparticles (TiO_2_, Al2O_3_, CuO, NiO and ZnO). At the end, future directions of zebrafish model and relevant assays to study nanoparticle toxicity have also been argued.

## Background

Now-a-days, nanotechnology encompasses an increasing impact on the industrial revolution accounting for multi-billion-dollar business sector. Various industrial sectors, including tissue engineering, drug delivery, imaging, diagnostics, surface texturing, and bio-interfaces are currently using nanomaterials in their products [1, 2]. Hence, with the growing business impact of nanotechnology, Business Communications Company (BCC) projected that the nanotechnology industry was approximately 7.6 billion USD market in 2013 which further has a potential to rise up to 1 trillion USD by 2020 [3]. Nanotechnology is upcoming as a solution across a range of industrial problems and also acts as a crossroad for different enabling technologies like biotechnology, computational science, physical science, communications technology, cognitive science, and others [4, 5]. Mihail Roco of the U.S. National Nanotechnology Institute visualized four generations of nanotechnology [6] and expected that the third generation is about to appear around 2010 with different types of nanosystems and thousands of their interacting components. In accordance, until 2013, 1814 nanoparticles products are commercially available in the market [7].

Nanotechnology is known for the designing, development, description, and applications of materials at nanometer scale. Furthermore, engineered nanodevices are finding an ever-expanding range of applications due to the possibility of versatile modifications in their shape, size, surface, and chemical properties. The surface of nanomaterials can be modulated according to their application such as for drug delivery the biocompatibility of the nanomaterials can be modified and their cell specific targeting ability can also be enhanced by attaching them with targeting ligand [8, 9]. Presently, people are using a wide variety of commercially available nanoparticles for their daily utilities such as—(1) silver (Ag) nanoparticles are used in sheets and clothing, (2) titanium dioxide nanoparticles are used in different cosmetics, lotions and creams, (3) carbon nanoparticles are used in motorcycle and in bicycle, and (4) clay nanoparticles are used in beer bottles [10]. With the growing demand of nanoparticles and their commercial potential, the area of nanotoxicity has grown considerably during the last 10–15 years and we are hoping that in near future it will address our serious concern. The area of nanotoxicity also addresses the regulatory aspects for the growing explosion in nanoparticle technology. The enhanced possibility of nanoparticle exposure and their toxic effects on consumers and environment is also a thoughtful issue to be highlighted [11].

**Fig. 1:**
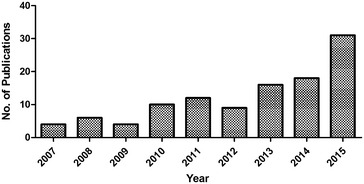
Increasing trend in the publications in zebrafish research (2007–2015). Keyword (“nano” and “zebrafish”) searched was performed from PUBMED, NCBI database. Search was conducted on 14th July 2016

In fact, there are an increasing number of literatures that documents the concern over toxicity for broad range of engineered nanoparticles/nanomaterials such as CNT, fullerenes, graphene metal nanoparticles, metal oxides nanoparticles, crystalline materials, amorphous materials and nano-sized polymers [12]. Various toxicity experimental assays/model organisms are used time to time for this purpose, such as in vitro cellular assays, multi-cellular model organisms (such as daphnia and sea urchin) [13] and higher animal models like mice [14]. With the use of the compound material from in vitro to in vivo experiments, it has been observed that the higher animal models are more valuable in comparison to simple experimental models. Each and every year, the number of engineered nanomaterials and their products are continuously increasing and there is a critical need to develop representative model organism, able to assess nanotoxicity accurately and to screen the nanoparticles at throughput level. In this regard, zebrafish as an in vivo model organism has attracted scientific interest because of its unique features (Fig. 1).

In this depth review, firstly we focused on the advantages of zebrafish that makes it a popular experimental animal model for various studies. Further, we described the different nano-toxicological assessment methods in detail. Afterwards, various nanomaterial toxicological studies conducted using zebrafish model have been emphasized. Finally, we also highlighted the drawbacks and future prospects of zebrafish model for nanotoxicity studies.

## Zebrafish: a popular experimental animal model

**Fig. 2:**
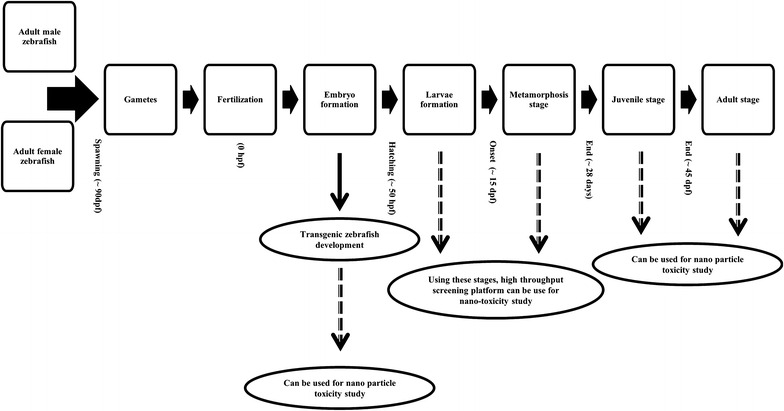
Schematic diagram describes—the different stages of zebrafish development and their relevancy for nanotoxicity study

The global acceptance of zebrafish as a modern experimental animal model is increasing gradually. This animal model is becoming popular in the fields of toxicology and biomedical research during both adult as well as embryonic stages. The reason for this wide recognition of zebrafishes as popular animal model is due to some exceptional set of characteristics it possesses. Some of them are their small size, very high reproducibility, quick development, transparency of the embryo and acquiescent to genetic as well as chemical screens [15, 16]. Additionally, we can also find extensive literature on zebrafish experiments. It has been noted that zebrafish is a small sized animal and, therefore, it can be handled without any difficulty. The eggs hatch rapidly, and the larvae can start feeding after 120 h of fertilization indicating the onset of experiments on zebrafish larvae from that point [17]. Another advantage is that the embryos are transparent, and all cells are observable since early larval stages. In addition, organs and tissues may also be readily visualized in vivo and can be examined instantly [18, 19]. Furthermore, zebrafish is known to possess high fecundity rate generating large number of embryos. As an example, the females spawn around 300 eggs per week under ultimate conditions. This may result in fecundity of >300,000 eggs per kg of the female. Additionally, it can spawn in the laboratory aquarium by adding flora and gravel into the tank [20, 21]. It has also been observed that the eggs hatch rapidly and organogenesis occurs quickly. As a result, the major organs are developed within 5–6 days post-fertilization (dpf) in larvae (Fig. 2). At the average of 350 dpf, females can attain size of 38 mm while males can attain a maximum mean size of 35 mm with a weight of 0.9 and 0.6 g, respectively [22]. Another major advantage of zebrafish is that the cardiovascular, nervous and digestive systems of this model animal are similar to mammals [20]. In addition, highly conserved signaling pathways are found both in zebrafish and humans with a high level of genomic homology [23]. Thus, genetic analysis assessment of a particular gene function by transgenic development and knockdown experiments can also be performed through zebrafish with an ease [24]. Recently, National Institutes of Health (NIH), USA, has started to encourage the zebrafish model organism for the analysis of different diseases with a genetic program [25]. Zebrafish genetic map has also been developed, showing >2000 microsatellite markers and 400 distinct genes. As observed, high level of resemblance exists among the human and zebrafish genomes (more or less 75 % similarity) making it a feasible animal model for analytical studies [26].

## Different model organisms and uniqueness of zebrafish model to understand the toxicity

Several model organisms are been explored to understand the mechanism of various human diseases along with their genetic disorders. Frequently used organisms are yeast (*Saccharomyces*) [27], Drosophila [28], *Caenorhabditis elegans* (*C. elegans*) [29], zebrafish [30], mouse [31], monkey [32], and many more. Drosophila or *C. elegans* are outstanding models for studying the events of genetic functions related to the common molecular machine. These models help us to understand regulatory molecular machinery, such as a complex of communicating proteins or a signal-transduction pathway [33, 34]. On the contrary vertebrate model systems such as the mouse, zebrafish, and monkey are most preferred models for the human diseased state as compared to invertebrate model organisms. However, vertebrate model organisms are the most complex model systems among all of them. Both the advantages and disadvantages of all these animal model organisms are already discussed in previous reports [35, 36].

Among the entire vertebrate models the most widely studied is mouse model system. However in mouse model, some disadvantages are notable such as (1) “forward” genetics is difficult; (2) inside the mother embryonic manipulations and fetal experiments are complicated; (3) developmental stages, as well as life cycle, is moderately time-consuming; (4) high cost is involved for animal breeding and developing animal house facilities. As zebrafish offers various advantages over mouse model, it is emerging as an important model system which can connect development, disease, and toxicological studies. Henceforth, in the present scenario zebrafish can be represented as an interesting tool for assessing nanoparticle toxicity [20].

## Different assessment methods to evaluate nanoparticle toxicity

In respect to various advantages offered by zebrafish, as discussed above, it is a unique model from the view point of the environment and human safety (EHS). The information produced from nano EHS studies can help to manage the risk of nanomaterials and nanotechnology related products in near future. The information may also assist us in framing the effective guidelines on protective measures, quality controls, and design strategies for improving nanomaterials and minimizing toxicity [37–39]. Using this model organism, several specific protocols have been used for the toxicity screening which are as follows:

### Hatching achievement analysis

Zebrafish hatching is one of the most important events for the researchers to understand the chemical/nanomaterial toxicity. Various reports described the time span of zebrafish embryonic developmental stages, in detail. Acceding to Kimmel et al. [40] hatching occurs within the first 3 days. Furthermore, Villamizar et al. [41] illustrated that hatching event is related to a rhythmic pattern corresponding to the light phase. The maximum eggs are hatched at approximately 2 dpf and the left over hatches roughly at 3 dpf. The correlation of successful hatching efficiency and embryo toxicity is an important parameter to evaluate the nanotoxicity. Recently, researchers used TiO_2_ nano-particles to understand the hatching events and the embryo toxicity by evaluating the relationship between hatching success rate and hours post exposure. It was observed that TiO2 nanoparticles can cause premature hatching in zebrafish embryos, dose dependently [42]. Ong et al. [43] also presented a view insight of zebrafish hatching under the effect of nanoparticle. They reported complete inhibition of hatching and embryo death within chorion upon nanoparticle exposure. They also concluded that nanoparticles interact with the hatching enzymes and thus they are responsible for toxicity rather than their ionic forms. Also, Vogt et al. [44] analyzed the chemical toxicity using zebrafish embryos at 24–48 h post fertilization (HPF) in 96-well plates. In this assay, multi-well plates are used with zebrafish embryos with the addition of a small molecule (BCI) to analyze hyper-activation of FGF signaling .

### Developmental malformation analysis of embryos and organs

Malformation of embryos and organs is another parameter for toxicity screening. Developmental malformation includes incomplete organ development such as eye or incomplete body part development such as incomplete head formation and deformities of body parts such as bent notochord, fin malformation and lack of pigmentation. Ali and Legler [45] showed the non-lethal developmental malformations of zebrafish embryos after the exposure of chemical (nonylphenol) even at low dose. Moreover, Usenko et al. [46] evaluated of carbon fullerene [C_60_, C_70_, and C_60_(OH)_24_] toxicity using zebrafish embryos, in vivo. They observed caudal fin malformation at the concentrations of 200 ppb of C_60_, C_70_ and yolk sac edema, pericardial edema and pectoral fin malformations over the concentrations of 2500 ppb of C_60_(OH)_24_. Additionally, they also observed swelling of zebrafish embryo and delay in development upon exposure to 5000 ppb of C_60_(OH)_24_. Therefore, the use of zebrafish model can be proposed for screening the toxicity profile of nanomaterials and their rapid feedback.

### In vitro/in vivo imaging

Another important use of this model is to analyze the toxicity through in vitro imaging. Several specific types of imaging techniques can be used for toxicity study. For instance, dynamical cell imaging of zebrafish embryos is a method for safety assessment which is now used by several scientists [47]. Additionally, high-content imaging (HCI) methods also permit us to collect automated visual data as well as image analysis. The HCI method is very rapid and can be used for the chemical screening in zebrafish larvae [48]. Presently, whole-body imaging is a challenging method to understand the pathophysiology of 3D morphological structures. Using synchrotron X-ray micro-CT, whole-body imaging was performed for hypercholesterolemic female zebrafish to understand the pathophysiology of 3D morphological structures [49]. Furthermore, in vivo real-time imaging is also used to understand the toxicity as well as size-dependent transport of metal nanoparticles using zebrafish embryos. Although the commercialization of silver (Ag) nanoparticles is expanding, its toxicity is also well known [50]. In view of this, recently a bio-imaging study on the toxic effect of sodium cholate templated Ag nanoclusters during the developmental stages of zebrafish embryo was conducted [51]. Thus, due to the wide application of bio-imaging, zebrafish can be further explored to determine the toxicity profile of various nanomaterials.

### Transgenic zebrafish as live biosensor

Transgenic zebrafish model is a potential choice in toxicological studies and currently, it is also used to evaluate the toxicity as live biosensor (Table 1). After exposed to toxic chemicals, morphological changes are well noticeable in zebrafish [52]. Lee et al. [53] developed a transgenic line of zebrafish embryos entitled as “huORFZ embryos” which can accurately detect various kinds of pollutants compounds and can be further used as water alarm system to monitor the disposal of hazardous pollutants in the water bodies. The significance of cytochrome P450 (CYPs) as an important biomarker for detecting carcinogen compounds, such as polycyclic aromatic hydrocarbons (PAHs), is well reported. Therefore, Hung et al. [54] generated a transgenic line of zebrafish using a CYP-green fluorescence protein (CYP-GFP) construct for live imaging and to detect the water toxicity level using polychlorinated biphenyls (PCBs) as pollutant. They observed various morphological alterations in zebrafish upon PCB exposure suggesting the use of CYP-GFP as a live biosensor. Almeida et al. [55] also developed fluorescent transgenic zebrafish expressing a destabilized fluorescent protein (DsRED) to understand the toxicity generated by pesticide i.e. methyl parathione. Their observations also suggested the use of transgenic zebrafish as a potential live biomarker for toxicity analysis.

**Table 1:** Different types of transgenic zebrafish used to the nanoparticle/chemical toxicity study

	Nanoparticle/chemicals used	Transgenic zebrafish type that is used to understand the nanoparticle toxicity	Remark	References
1.	TiO_2_, SiO_2_, CuO	Transgenic (nacre/fli1:EGFP) zebrafish	Transgenic fish exposed to TiO_2_,SiO_2_, CuO particles with a concentration of 0.01, 1 and 100 μg/ml concentrations and noted that CuO nanoparticles inhibit vasculogenesis	[140]
2.	TiO_2_, C60(OH)24) (hydroxylated fullerenes/)	Gene expression of zebrafish embryos	Circadian rhythm gene/(s) deregulated by nanoparticles	[141]
3.	TiO(2)	ARE transgenic zebrafish	Exposure TiO(2) nanoparticle cause death of zebrafish embryos	[137]
4.	Small molecules	Transgenic embryos expressing green fluorescent protein in myocardium	Small molecule alter in heart rate of transgenic embryos	[142]
5.	Metal oxide nanoparticles such as CuO, ZnO, NiO, and Co(3)O(4))	hsp70:eGFP transgenic zebrafish larvae	CuO, ZnO, and NiO may augmented expression of hsp70:eGFP in transgenic zebrafish larvae	[136]
6.	Inorganic nanorods	Transgenic (fli1a:EGFP) zebrafish embryos	This study noted that—ROS mediated angiogenesis in transgenic zebrafish embryo using inorganic nanorods	[143]
7.	Cadmium selenium (CdSe) quantum dots (QDs) coated with mercaptopropionic acid (MPA)	FLI-1 transgenic zebrafish larvae	abnormal vascularization occoured in transgenic zebrafish larvae	[144]
8	Mesoporous silica nanoparticles (MSNPs)	lysC:DsRED2 transgenic embryos	It can be used to deliver bioactive compounds	[145]

### Behavioral analysis

Interestingly, zebrafish behavioral response is also a sensitive indicator for abnormal change in toxicity and thus nowadays it has become a most important parameter to estimate the toxicity level [56]. Within the behavioral responses, swimming kinetics is the most relevant and highly studied parameter. It has been prominently recorded that swimming speed and depth were altered by the chemical toxicity [57]. Kokel et al. [58] generated a behavioral ‘barcode’ to understand the chemical toxicity. Behavioral abnormalities such as abnormal startle behavior following a tap stimulus after exposure to gold nanoparticles at 122 dpf was evaluated by Truong et al. [59]. Another experiment performed by Chen et al. [60] has also shown that TiO2 nanoparticles affect larval swimming parameters, including velocity and activity level.

### Disruption of gill, skin and endocrine system

The disruption of gills, skin and endocrine system by nanoparticles is another parameter to understand nanoparticle induced toxicity. It has been reported that gills are most important targets of waterborne objects such as waterborne nanoparticle. Silver ions (Ag^+^) generated by Ag nanoparticles are well documented for their acute toxicity, mainly due to their interaction with the gills. In the gills, ionic Ag^+^ inhibits Na^+^/K^+^-ATPase action and the enzymes related to Na^+^ and Cl^–^ uptake, finally affecting osmoregulation [61, 62]. Griffitt et al. [63] showed that Cu nanoparticles (insoluble forms) are extremely toxic to zebrafish and their suspensions may damage gills lamellae. In addition reports showed that nanoparticle are also toxic to zebrafish skin. Researchers have observed deposition in zebrafish skin upon exposure to nanoparticle like Ag-BSA. In the embryo skin, the nanoparticles enter through diffusion or endocytosis where they accumulate on the epidermis layer of the larvae and causes skin abnormalities through apoptosis [64]. Chemical exposure can also cause endocrine disruption in zebrafish. Tu et al. [65] described the endocrine disruption in zebrafish by chemicals and performed endocrine gene transcription analysis which resulted in the increased expression of the estrogen-responsive gene Vtg1. The results also showed that there is no effect on the expression of the ERα gene. Another experiment by Miao et al. [66] reported that the titanium dioxide nanoparticles increased the bioconcentration of lead, and directs the interruption of thyroid endocrine system in larvae of zebrafish.

### Immunotoxicity

Immunotoxicity is classified as the toxic effect of xenobiotics on the normal functioning of immune system through direct or indirect methods. Direct immunotoxicity causes immune suppression leading to reduced resistance against various diseases, such as cancer [67]. Immunotoxicity of nanoparticles has been demonstrated by several scientists. Various studies showed that nanoparticles (including metal oxide nanoparticles) modulate cytokine production by generating free radicals. Some nanoparticles have also been linked to allergic sensitization and can increase the tendency of asthma [68]. Jin and Zheng [69] reported that a toxic chemical like cypermethrin induces apoptosis and immunotoxicity in zebrafish (Danio rerio) suggesting the possible use of zebrafish in immunotoxicity studies. Furthermore, Zhuang et al. [70] described the enantio-selective differences in the developmental toxicity and immunotoxicity of pyraclofoson zebrafish model. Several reports also suggested the use of live zebrafish embryos for analyzing the immunotoxic property of various chemicals. In this regard, Xu et al. [71] used transgenic, albino or AB line, live zebrafish embryos to understand the immune-toxic effects of chemicals such as dibutylphthalate. However, more researches are needed on nanoparticle-based immunotoxicity analysis on zebrafish.

### Genotoxicity

Genotoxicity is described as the damage caused to genetic information inside a cell due to the occurrence of chemical agents which results in gene mutation, chromosomal alteration and DNA damage [72]. Genotoxicity is a main risk factor for long-term toxic effects such as carcinogenesis. Zebrafish model has been proposed to study the different chemical-induced genotoxicity using various techniques. Cambier et al. [73] studied genotoxic effects of cadmium on zebrafish through RAPD and RT PCR. Furthermore, genotoxic effects of gold nanoparticle were also assessed using RAPD-based methodology on zebrafish after exposure to gold NPs [74]. In accordance to the above mentioned result, Geffroy et al. [75] investigated the effects of nanoparticles on genotoxicity through RAPD-PCR genotoxicity test in the zebrafish at very low doses of gold NP (36–106 ng gold/fish/day) and resulted in significant alteration of genome composition. However, to date fewer reports are available for genotoxic analysis of nanoparticles on zebrafish and thus more extensive studies are required in this area.

### Neurotoxicity

Due to the contact with toxic substances, the nervous tissue gets damaged resulting in significant irregular activity of the nervous system, which is called neurotoxicity. These toxic substances to nerve cell are referred as neurotoxins [76]. Neurotoxicity of nanoparticles has been determined earlier from time to time suggesting that nanoparticles can reach the brain and can cause neurodegeneration [77, 78]. In vivo and in vitro experiments discovered that combustion-derived nanoparticles are neurotoxic, because of the incidence of nanoparticle aggregation [79]. Therefore, in view of applying zebrafish model to determine nanoparticle neurotoxicity the radioprotective effect of dendrofullerene nanoparticle (DF-1), a C_60_ fullerene derivative, has been assessed in zebrafish embryo to understand the neurotoxicity resulting in dose-limiting toxicity level [80].

Sheng et al. [81] also evaluated TiO_2_ nanoparticle-induced neurotoxicity using zebrafish model. It has been reported that TiO_2_ nanoparticle significantly activated expressions of different genes such as BDNF C-fos and C-jun. Conversely, this NP suppressed the expressions of genes such as p38, NGF, CRE resulting in the brain damage of zebrafish. Such contradictory observations provide a requirement for evaluating the nanoparticle caused neurotoxicity in future studies.

### Reproductive toxicity

Reproductive toxicity is also among the important parameters of nanoparticle toxicity measurement [82]. Zebrafish model is one of the best models to assess the reproductive toxicity due to its high reproduction rate. As observed, nanoparticle affects male and female reproductivity and fetal development. Wang et al. [83] assessed the disturbance in zebrafish reproduction after the chronic exposure of TiO_2_ nanoparticles. Their study showed 9.5 % decrease in the collective number of zebrafish eggs after 13 weeks of TiO_2_ nanoparticle exposure.

### Mortality assessment

Acute exposure to nanoparticles enhances the chance of critical illness as well as increase in mortality rate. Duan et al. [84] treated zebrafish embryos with silica nanoparticles in their experimental model system and observed enhanced embryonic mortality. ZnO is another toxic nanoparticles which caused hatching delay, skin ulceration and high mortality of zebrafish [85]. It has been illustrated that a relationship exists between the shape of the nanoparticles and the mortality incidences. Therefore, Hua et al. [86] evaluated the toxic effects of differently shaped zinc oxide nanoparticles and observed that ZnO nanosticks are more toxic as compare to other NPs resulting in increased mortality and reduced hatching in zebrafish. Polyamidoamine (PAMAM) is one of the significant dendrimers containing a diamine which is used as versatile precursor for dendrimer-based delivery of active pharmaceuticals [87]. Pryor et al. [88] showed 100 % mortality in zebrafish at 24 hpf by PAMAM dendrimers illustrating its toxic effects.

### Other methods

Chemical toxicity screening in zebrafish model has been performed using both larval stage as well as adult zebrafish [89]. Hermsen et al. [90] developed zebrafish embryo toxicity (ZET) test to analyze embryo- toxicity caused by two classes of chemicals in a modified zebrafish embryo. In their study, toxicity level of six 1,2,4-triazole antifungals and eight glycol ethers were evaluated. Among glycol ether metabolites methoxyacetic acid and ethoxyacetic acid and among triazoles flusilazole were most potent in retarding the growth and inducing malformation in zebrafish. Another newly developed computational analysis has also been used by Toxicology Research Program of the U.S. EPA to understand the toxicity of the chemical library. Through the experiments, researchers evaluated the toxicity of the 309 ToxCast™ Phase I chemicals using a zebrafish screen [91]. In a study, whole male adult zebrafish was exposed to high doses of nickel chloride, cobalt chloride or sodium dichromate for 24 h at various concentrations corresponding to their respective 96 h LC values and was evaluated for alterations in gene expressions. Histopathological changes were observed for each metal in their target specific organs and tissues signifying the role of adult zebrafish as a robust model in toxicogenomics [92].

## Nanotoxicology in zebrafish

Nanotoxicology is an interdisciplinary field, cross-linking various subjects such as chemistry, physics, biology, medicine and toxicology [93, 94]. Though, this area has many applicative approaches yet it is still in nascent form and the majority of the nanotoxicology studies are confined only within in vitro models. Some research groups performed toxicological studies using animal models (in vivo) which are supposed to be significant due to the diversity in animal physiology and anatomy in animal models. Recently, for this purpose zebrafish is proposed as one of the most successful model and notable advancement has been made in nanotoxicology studies using zebrafish as animal model [95]. This section categorizes some of the currently available toxicity data of several nanoparticles which has been studied using zebrafish model.

### Silver nanoparticle

Silver nanoparticles are the most extensively studied nanoparticles. They are also widely used as therapeutic agents [96], as antimicrobial agents [97], in drug delivery systems [98], as biosensors [99] and in cosmetics [100]. Size-dependent toxicity of AgNP was observed indicating that the size of the NP is one of the important factors for their toxicity profile. Lee et al. [101] performed in vivo quantitative study and demonstrated size-dependent transport and toxicity of Ag-NPs in zebrafish. In their study, they showed that Ag nanoparticle in size range 30–72 nm diameters were able to diffuse into the zebrafish embryos through chorionic pores via random Brownian motion and thus they can show more potent toxic effect. On the contrary, Bar-Ilan et al. [102] synthesized AgNP in different size range (3, 10, 50 and 200 nm) and treated them to zebrafish embryos rearing container. They observed that there were size independent 100 % mortality incidences after 120 hpf. Hence, the size dependent toxicity profile of AgNP is still debatable. Surface defect is another parameter of nanoparticle toxicity. George et al. [103] verified the toxic effect and surface defect caused by Ag nanoparticle in both fish cell lines and zebrafish embryos. Furthermore, in a recent study George et al. [104] also showed the effect of solar light in increasing the toxicity of Ag.

The charge dependent transport and toxicity of peptide-functionalized Ag-NPs into early developing stage zebrafish embryos was also demonstrated. A recent study concluded that Ag-peptide NPs are much more biocompatible than the citrated Ag NPs [105]. Choi et al. [106] evaluated oxidative stress and apoptosis in zebrafish liver and concluded the hepatotoxic behavior of AgNPs. They also observed the disruption of hepatic cell cords and apoptotic changes in Ag nanoparticles exposed zebrafish due to the upregulation of p53-related pro-apoptotic genes such Bax, p21 and Noxa. The influence of AgNPs on the neurological development of zebrafish was studied by Xin et al. [107].They showed that the introduction to AgNPs can alter the neurological development and can result in small head along with the presence of hypoplastic hind brain, little eye, and cardiac defects [107]. In view of the toxicity profile of AgNP, some recent studies proposed that the chemical transformations of Ag nanoparticles can be an important way to slow down the nanoparticle toxicity. For instance, Devi et al. [108] proved that sulfidation method can delay AgNP toxicity in zebrafish model. Furthermore, biosynthesis of silver nanoparticles can also be a better way to reduce its toxicity level [109].

### Gold nanoparticle

Nanoscale gold particles have attained massive scientific interest due to their chemical stability and unique optical properties [110]. Gold nanoparticles have shown great potential in medical industry such as therapeutic agents, as photothermal therapeutic agent, as drug carriers, etc. [111]. Presently, gold nanoparticles are used as promising agents for cancer therapy [112], as imaging particles in optical microscopy [113] and confocal laser microscopy [114]. It is also used in diagnostics such as dot immunoassay, immunochromatography, etc. [115]. Today, gold nanoparticles are among the most widely studied nanoparticles.

Like other metal nanoparticle, cytotoxicity of gold nanoparticles in humans has also been reported [116, 117]. Therefore, to understand the in vivo toxicity profile of gold nanoparticles some studies used zebrafish as in vivo animal model. Kim et al. [118] analyzed the toxicity outcomes of cationic ligand functionalized gold nanoparticles in the development of zebrafish embryos including lethality and other morphological defects. As observed, the functionalized gold particles caused abnormalities in the eye development and affected pigmentation in eyes leading to behavioral and neuronal damage. Harper et al. [119] assessed the toxicity mapping of AuNPs of different sizes and surface charges (such as neutral, positive and negative surface charges) using an embryonic zebrafish model. They concluded that the mortality and other developmental disorders are closely related to the morphological and chemical characteristics of gold nanoparticles signifying of the importance of controlled synthesis of nanomaterials. In another similar approach, toxic effect of gold nanoparticles possessing three different functional group with different surface charges on zebrafish embryos was assessed exhibiting hypo-locomotor activity and abnormal behavioral activity [59]. Real time in vivo imaging was further used in a different study to determine the size-dependent transport and toxicity of AuNPs in zebrafish embryos which revealed the presence of AuNPs inside the embryos throughout their whole developmental duration [50]. In addition, Dedeh et al. [74] exposed zebrafish with AuNPs for 20 days at two concentrations i.e. 16 and 55 µg/g dry weights and recorded the alterations in oxidative stress, mitochondrial metabolism and neurotransmission.

### Carbon nanotubes (CNT)

Carbon nanotube showed numerous distinctive chemical and physical characteristics which make it equivalent to the biological macromolecules such as antibodies, enzymes, plasmid DNA, etc. CNTs have become a promising candidate for the delivery of chemotherapeutic agents such as paclitaxel and doxorubicin, small interfering RNA (siRNA), different biological molecules, genes, vaccines and antibodies. This is due to the fact that because of high surface area it can conjugate with a wide variety of therapeutic molecules [120, 121]. However, the toxicity of carbon nanotube is an upcoming and significant challenge nowadays [122]. The toxicity reports explaining the potential impact of CNT’s on human health and the environment are very inadequate and thus it requires more studies through improved methodologies. Ali-Boucetta et al. [123] discussed the pitfalls in traditionally used in vitro models for toxicological studies and highlighted the need for more reliable model systems. They also performed MTT and the LDH assays to comprehend the interaction of CNT with cell cultures and to understand the cytotoxicity of CNT’s. Luanpitpong et al. [124] described the effects of CNT on lung and dermal cellular behaviors which showed the potential pulmonary and dermal risk associated with CNT exposure.

Recently, using zebrafish model bioaccumulation and distribution of multiwalled CNTs was evaluated which showed the bioaccumulation factor of 16 L/kg fish wet weight [125]. Another study performed by Li et al. [126] revealed CNT-induced biochemical alterations in zebrafish. They further demonstrated that CNT exposures can stimulate the brain and gonadal alterations. In a different study, the toxicity level of functionalized CNT’s with different lengths was evaluated in zebrafish embryo and was further suggested that length of CNT’s also plays a major role in their toxicity profile, in vivo [127]. In a study by FilhoJde et al. [128] CNT network pellets having agglomerated multi-walled CNTs (MWCNTs) with a standard diameter about 500 nm were not found genotoxic to zebrafish model. However, they observed reversible inflammation in the zebrafish gills.

Besides MWCNTs, the malformations and mortality induced by C_60_ fullerene was also studies in zebrafish embryo. It was observed that C_60_ fullerene causes dose dependent necrotic and apoptotic cell death in the head and trunk of zebrafish embryo. Although in comparison to C_60_, C_60_(OH)_24_ was found less toxic to the head region. Further, it was demonstrated that C_60_ causes oxidative stress and was depicted as the main reason for malformations in zebrafish embryo [46].

### Metal oxide nanoparticles

Metal oxide nanomaterials are commercially used in different emerging areas such as information technology, energy storage, medicine, and catalysis [129, 130]. Several types of metal oxide nanoparticles are used in industrial production of nanoparticles such as TiO_2_, ZnO, Fe_3_O_4_, Al_2_O_3_, and CrO_3_. Jeng and Swanson examined the toxicity of metal oxide nanoparticles where they observed apoptotic behavior of ZnO nanoparticles in cells [131]. The toxicity of metal oxide nanoparticles was also assessed through zebrafish model. Zhu et al. [132] evaluated the toxicity of three metal oxide nanoparticles (TiO_2_, Al2O_3_ and ZnO) using zebrafish embryos. They observed tissue ulceration in zebrafish larvae exposed to aqueous suspensions of nZnO and ZnO/bulk. Additionally, they showed that nTiO_2_, nZnO and nAl_2_O_3_ exerted toxic effects on both zebrafish embryos and larvae. An attractive study was performed by Palaniappan and Pramod to compare the effect of bulk and nano TiO_2_ on zebrafish brain by using FT-IR technique where nTiO_2_ proved to be more toxic as compared to bulk TiO_2_ [133]. Similar results were again observed by Palaniappan and Pramod while investigating the effect of nTiO_2_ and bulk TiO_2_ on zebrafish by FT-Raman Spectroscopy [134]. Additionally, Chen et al. [135] also explored that nano TiO_2_ causes toxicity effects to zebrafish model, and the chronic toxicity of TiO_2_ NP is concentration as well as time dependent. These nanoparticles get distributed and accumulate in different body parts of zebrafish such as gill, liver, heart and brain. Compared to other nanoparticle, TiO_2_ NPs can also cross blood–brain barrier [78]. Transgenic zebrafish is an important line which helps to detect the metal oxide toxicity. It has been observed that some other dissolvable metal oxide nanoparticles such as CuO, ZnO and NiO hinders in hatching process of transgenic zebrafish embryos line generated using hsp70 and eGFP genes [136]. Bar-Ilan et al. [137] used a transgenic zebrafish line (ARE:eGFP) for detecting the generation of oxidative stress in the larvae upon TiO_2_ nanoparticles exposure. The experiment explains that TiO_2_NPs absorbs photons and produce electron–hole pairs that interact with water and oxygen to form cytotoxic ROS. Finally, researchers concluded that the TiO_2_ NPs exposure to zebrafish embryos leads to malformation and death.

## Drawbacks of zebrafish model for nanotoxicity study

To evaluate the toxicity profile of nanomaterials, zebrafish model was used in various studies as an in vivo model system. The toxicity level of these nanoparticles was assessed by observing the malformations and functional defects in zebrafish. However, it is very clear from the literature survey that nanomaterial based immunotoxicity assay is still lacking. Additionally, due to the rapid developmental stages in zebrafish it is very difficult to perform systematic embryo-based nano-toxicity assays. However, automation and advanced technologies can help in nano-toxicity screening with zebrafish embryos. Several nanomaterials are used for the therapeutic purpose such as drug delivery and antimicrobial therapeutics. Therefore, it is needed to understand the absorption, distribution, metabolism and excretion (ADME) properties of these nanomaterials. However, it is ambiguous that how ADME assay will perform in zebrafish model after nano-drug delivery.

## Future prospects

Zebrafish has shown its enormous potential as an in vivo model for nanomaterial toxicity study. Presently, various molecular biology techniques and zebrafish model transgenic lines have been developed for this purpose. Various zebrafish microarrays and huge genomic resources are nowadays available for nanotoxicity evaluation. All these advance resources makes zebrafish an extremely multipurpose system for toxicogenomic studies of nanomaterial in the very near future. Proteins and genes expression studies of zebrafish development have the huge possibility to uncover the still debated nanomaterial toxicity. Although, high throughput screening systems using larval stages of zebrafish is already being exploited for nanomaterial toxicity study, there is still an enormous potential for nanomaterial toxicity assays.

## Conclusion

Presently, zebrafish have become a smart vertebrate model for toxicological testing. In Germany, the zebrafish embryo test was introduced as a standardized ISO program in the evaluation of chemicals. This assay can be used for water testing to evaluate the level of environmental contaminants [138]. Furthermore, this animal model is much faster, cheaper and more efficient animal model for more than a decade [139], and this model is used for toxicological testing of nanomaterial. With the use of modern technology, the zebrafish might be able to become a significant alternative of other mammalian models for toxicological testing of nanomaterial in forthcoming years.

## Abbreviations

hsp70: heat shock protein 70
eGFP: enhanced green fluorescence protein
ROS: reactive oxygen species
DsRED: zebrafish transgenic lines that express green or red fluorescent proteins
lysC: lysozyme C
FLI-1 gene: friend leukemia integration 1gene
